# Phenotypes of lymphocytic alveolitis in our group of bronchoalveolar lavage fluids

**DOI:** 10.1186/1939-4551-8-S1-A117

**Published:** 2015-04-08

**Authors:** Gustav Ondrejka, Andrea Ondrejkova

**Affiliations:** 1 Hospital Novy Jicin, Czech Republic; 2 Agel Laboratories, Czech Republic

## Background

Bronchoalveolar lavage (BAL) has a stable place in the diagnostics of various forms of the diffuse parenchymal lung disease. The analysis of cellular components of the bronchoalveolar lavage fluid (BALF) informs us about inflammatory and immune processes in the alveolar space. This can be very helpful in the diagnostics of many interstitial lung diseases (ILDs), but also in the diagnostics of pulmonary haemorrhage and bronchoalveolar carcinoma or lymphomas. The aim of study is to present our experience in cellular analysis of BALF.

## Methods

Lymphocyte subsets were determined by flow cytometry. We utilized monoclonal antibodies directed against the CD3, CD4, CD8 (T-lymphocytes), CD19 (B-lymphocytes), CD16 (NK cells), and HLA-DR antigen to determine the degree of lymphocyte activation.

## Results

Over the past 10 years (2003–2013) we have analysed 4540 BALFs. We evaluated total cell count, immunocompetent cells profile and phenotype of lymphocytes.

An increased percentage of lymphocytes (above 15%) were found in 1680 BALFs (37%). The phenotypes of these cases of lymphocytic alveolitis were determined via monoclonal antibodies as mentioned above. CD3+T-lymphocytic alveolitis was established in 1677 BALFs (99.8%). This represents an overwhelming majority of BALFs with elevated lymphocyte count in patients with interstitial lung diseases. CD19+B-lymphocytic alveolitis was found in 2 BALFs only. These interesting accidental findings resulted in the establishment of B-lymphocyte hematopoietic infiltrative neoplasm in patients suspected of interstitial lung diseases. CD16+NK cells alveolitis was found in 1 BALF only of a patient with histologically confirmed pulmonary fibrosis type of UIP.

## Conclusions

We concluded that lymphocytic alveolitis is a nonspecific pathological finding to be followed by detailed lymphocyte phenotype analysis. Owing to the fact that the phenotypes of an overwhelming majority of the cases of lymphocytic alveolitis in our group (99.8%) were CD3+, we conluded that T-lymphocytes almost exclusively persist within the alveolar structures and play an important role in the pathogenesis of many interstitial lung diseases. Consequently, monoclonal antibodies directed against the CD3 antigen (T-lymphocytes), against the CD4 and CD8 antigens (T-lymphocytes subsets), perhaps even the antibody against the HLA-DR molecule (the T-lymphocyte activation marker) are sufficient for routine BALF lymphocyte phenotype analysis.

